# Hidden in the Middle: Culture, Value and Reward in Bioinformatics

**DOI:** 10.1007/s11024-016-9304-y

**Published:** 2016-07-11

**Authors:** Jamie Lewis, Andrew Bartlett, Paul Atkinson

**Affiliations:** School of Social Sciences, Cardiff University, Glamorgan Building, King Edward VIIth Avenue, Cardiff, CF10 3WT UK

**Keywords:** Bioinformatics, Culture, Reward, Values, Interdisciplinarity, Disciplines

## Abstract

Bioinformatics – the so-called shotgun marriage between biology and computer science – is an interdiscipline. Despite interdisciplinarity being seen as a virtue, for having the capacity to solve complex problems and foster innovation, it has the potential to place projects and people in anomalous categories. For example, valorised ‘outputs’ in academia are often defined and rewarded by discipline. Bioinformatics, as an interdisciplinary bricolage, incorporates experts from various disciplinary cultures with their own distinct ways of working. Perceived problems of interdisciplinarity include difficulties of making explicit knowledge that is practical, theoretical, or cognitive. But successful interdisciplinary research also depends on an understanding of disciplinary cultures and value systems, often only tacitly understood by members of the communities in question. In bioinformatics, the ‘parent’ disciplines have different value systems; for example, what is considered worthwhile research by computer scientists can be thought of as trivial by biologists, and *vice versa*. This paper concentrates on the problems of reward and recognition described by scientists working in academic bioinformatics in the United Kingdom. We highlight problems that are a consequence of its cross-cultural make-up, recognising that the mismatches in knowledge in this borderland take place not just at the level of the practical, theoretical, or epistemological, but also at the cultural level too. The trend in big, interdisciplinary science is towards multiple authors on a single paper; in bioinformatics this has created *hybrid* or *fractional* scientists who find they are being positioned not just in-between established disciplines but also in-between as middle authors or, worse still, left off papers altogether.

## Introduction

The Human Genome Project (HGP), the accomplishment of which involved increasingly automated and ‘industrialised’ laboratory techniques and the integration of the skills required to handle large amounts of data, catalysed a change in the way in which biology is conducted (Kay 2000; Liebler 2002; Stevens 2011). Organizationally, there is now a new model for approaching questions of biology; that of large-scale collaborative projects (Collins, Morgan, and Patrinos 2003). ‘Big science’ (cf. de Solla Price 1965; Galison and Hevly 1992) and ‘big biology’ (Bartlett 2008; Hilgartner 2013; Vermeulen, Parker and Penders 2013) are phrases used to characterise increases in the size, scale and scope of (biological) science that are accompanied by changes in scientific practice. These organizational transformations have been attended by a conceptual change too. In the post-HGP life-science there has been a shift from hypothesis-driven science to nominally ‘hypothesis-free’ science (Cooke Bailey, Pericak-Vance and Haines 2014). This change in orientation has been mirrored by a shift in the locus of practice, from the ‘wet lab’ (working in ‘traditional’ laboratories with biological material) to the ‘dry lab’ (working with data on computers).

A decade ago, in the wake of the accomplishment of the HGP, Diamond and Woodgate (2005) described “the move from genomics to post-genomics research”. They wrote of a “diversifying field”, in which “interdisciplinarity becomes increasingly important” as “traditional disciplinary boundaries become blurred, or break down, in the face of newly emerging sciences” (p. 239). This is the backdrop to this paper; a (life) science in which there has been a proliferation of new specialisms, many predicated on interdisciplinarity, colonising and exploiting the spaces – and synergies – between established disciplines. For example, Penders *et al.* (2008) have written of the way in which work in the ‘moist zone’, the space in-between the wet and dry laboratory, reveal the epistemological differences between these ways of working and how these differences can be overcome. The focus of this paper is specifically on bioinformatics; a marriage between the more traditional, established families of computer science and biology (Ouzounis and Valencia 2003; Lewis and Bartlett 2013; Stevens 2013; Bartlett, Lewis and Williams 2016). We argue that bioinformatics can be conceived as a borderland between cultures, a place where difference makes values – and the differences between values – explicit.

Although the term bioinformatics extends back to the middle of the twentieth century (Ouzounis and Valencia 2003; Suarez-Diaz 2010; Garcia-Sancho 2012; November 2012), the specialism experienced explosive growth following developments in computing (Stevens 2013) and the commencement of the HGP in the 1990s (Howard 2000; Lewis and Bartlett 2013). These advances created space for a new type of skilled interdisciplinary researcher – whose disciplinary hinterland may not necessarily have been the life sciences – to work on big biological projects. The increased interdisciplinarity of post-HGP life sciences was anticipated by those central to the project:

“The HGP has created the need for new kinds of scientific specialists who can be creative at the interface of biology and other disciplines, such as computer science, engineering, mathematics, physics, chemistry, and the social sciences. As the popularity of genomic research increases, the demand for these specialists greatly exceeds the supply […] There is an urgent need to train more scientists in interdisciplinary areas that can contribute to genomics” (Collins *et al.* 1998: 688).

That the importance of interdisciplinary specialisms such as bioinformatics will increase is clear; the collection of biological data has become increasingly routinized, automated, and large-scale – even if the possible uses of the data are not yet as obvious – as scientists turn the new tools of the post-HGP era to the challenges of more complex biological processes (see Brusic 2007; Suarez-Diaz 2010; Leonelli 2012; Marx 2013).

Beyond the shift in size, scope and practice of biology, the intellectual reorganisation of biology can also be expected to be accompanied by an institutional reorganisation for the academy. To quote Klein (1996): “interdisciplinarity is on everyone’s agenda, actually implementing it in institutional settings is a more difficult proposition” (p. 209). In what ways, for example, is an interdisciplinary field such as bioinformatics integrated into the existing structures of United Kingdom (UK) academic life? How do these new kinds of specialists fit amongst existing disciplinary categories? Does the creation of an interstitial domain disrupt the underlying ‘cosmology’ of academic knowledge-domains?

The focus of this paper is on the experiences of those who occupy this in-between space, and the ways in which they and their collaborators understand their place in the structures of the academy. We examine this space from the point of view of cultural values, and the way in which the practical impacts of working in an interdisciplinary space has as much to do with mismatched value systems as it does with mismatched scientific knowledge. How do those socialised into the value system of one discipline understand working in an area governed by a different set of disciplinary values? In what ways do such scientists feel incorporated into the reward and recognition system – i.e. the system of determining what is valued – of the life sciences? The answers to these ‘soft’ cultural questions can have ‘hard’ impacts.

## On Disciplinarity and Interdisciplinarity

Disciplines “are the intellectual structures in which the transfer of knowledge from one generation to the next is cast; that is they shape the entire system of education” (Weingart and Stehr 2000, p. xi). In universities and other scientific institutions, disciplines constitute the modern social order of knowledge, organizing knowledge (Gass 1979; Weingart and Stehr 2000), producing standards (Whitley 2000), policing behaviours (Krishnan 2009) and providing the conditions for reproducing the next generation of academic researchers. This is not to suggest that disciplines are natural kinds, or that they are immutable. But they are distinctive territories between which there are boundaries that are more or less porous. The social and institutional characteristics of these territories have an effect on the epistemological properties of the knowledge produced by the native ‘tribe’ (see Cook-Deegan 1994; Becher and Trowler 2001). This, in turn, further distinguishes one disciplinary territory from another. Disciplines and the researchers who constitute those disciplines therefore have a reflexive relationship. Disciplines configure those who work in that specialism, whilst the characteristics of what constitutes the discipline is shaped by those who make-up the field. The academic division of order is, naturally enough, reproduced in the social structure of the higher education system.

Interdisciplinarity, on the other hand, in its most general term, arises when a problem or task falls in between these disciplinary territories, with the resolution requiring the expertise of two or more disciplinary territories (Moran 2001). Interdisciplinarity can be imagined in two forms: ‘individual’ interdisciplinarity, in which a scientist masters two or more disciplines (Calvert 2010), and ‘collaborative’ or ‘collective’ interdisciplinarity, in which a group consisting of experts from different disciplines work together (Calvert 2010; Lewis and Bartlett 2013). Disciplinarity and interdisciplinarity imply one another: disciplinarity implies the existence of bounded disciplinary fields on which research can draw and from which expertise may be recruited; while, the very notion of interdisciplinarity is predicated on the idea of real difference across disciplinary boundaries.

While interdisciplinarity is increasingly seen as a virtue in and of itself by funders and administrators, interdisciplinary research has the potential to place projects and people in anomalous categories with regards to the established order of academia. Disciplinary boundaries may not mirror an order inherent in nature, but disciplinary boundaries have real effects. They define the social organisation of universities, the funding of research, the distribution of rewards (both material and symbolic), and the academic socialisation of students and researchers (Bourdieu 1988; Lewis 2010). Activities such as the Research Assessment Exercise (RAE) in the UK and its successor the Research Excellence Framework (REF) are also based on disciplinary classifications of research. Thus, if researchers do not fit into the existing categories, they may find that their interdisciplinary position of being in-between has repercussions that affect their careers, with opportunities for personal reward and professional recognition limited.

To repeat, though, the boundaries of disciplines are not immutable. As Gieryn (1983) writes, “science is no single thing: its boundaries are drawn and redrawn in flexible, historically changing and sometimes ambiguous ways” (p. 781). New fields emerge or are deliberately created, whether through ‘top down’ initiatives or by way of ‘bottom up’ efforts of researchers carving out a new territory. For example, the creation and support of multidisciplinary research programmes may necessitate *ad hoc* arrangements that span the divisions between disciplines. The ‘fashion’ for interdisciplinary research therefore can present disciplinary boundaries and differences as negatives; as the relics of a past age of academic life, as ivory silos of knowledge and expertise, as barriers to creativity and collaboration (Lowe and Phillipson 2009). Instead, the appropriate form of contemporary academic life is imagined as one that is defined in terms of shared problems and the collaborative search for solutions (Tait and Lyall 2007). In the UK, these interdisciplinary domains are often framed in terms of topics or ‘challenges’, such as the Research Council United Kingdom’s multidisciplinary research priority areas; ‘digital economy’, ‘energy’, ‘global food security’, ‘global uncertainties (security)’, ‘living with environmental change’, and ‘lifelong health and wellbeing’ (RCUK 2014). Of course, much of the work done in these programmes will be resolutely unidisciplinary in practice. For the work that is not based in one discipline, though, practical problems can arise. Existing structures are not well suited to judging – and assigning value to – work that is in-between established disciplinary territories.

In biology, the move to big(ger) science and the growing importance of work conducted in the ‘dry’ lab has opened up both the intellectual and institutional space for interdisciplinary work of this type. But what of the academics in this new age of pan-disciplinary problem solving? The creation of interstitial knowledge domains implies the emergence of a new type of academic; a type that may be viewed as hybrid, fractional, or even anomalous, in so much that this type does not conform to existing classifications. The experiences of scientists working in bioinformatics, as described in this paper, highlight the issues that accompany the interstitial in-betweenness of interdisciplinarity work in contemporary UK academic institutions, and the unease that many feel with regard to the way in which their work is recognised, valued, and rewarded.

## Defining Bioinformatics: A Research Context

This paper provides an example of the dynamics of scientific (inter)disciplines, specialisms, careers and reward structures. Previous work has discussed the tensions at play in bioinformatics (see Lewis and Bartlett 2013; Penders *et al.* 2008). Here, we build on this work to produce an account of this interdisciplinary space as an inter-cultural borderland in which differences in value systems, not just interests, are made explicit. We draw from two research projects conducted between 2004 and 2011 examining the status of UK academic bioinformatics. According to Ouzounis (2012), this period spans the shift in bioinformatics development from ‘Adolescence’ (2002–2006) to ‘Adulthood’ (2007–2011). There have undoubtedly been changes in bioinformatics during this time. These changes have been both external and internal, and among the internal changes there has been a generational differentiation (Bartlett, Lewis and Williams 2016). By this we mean that those of the ‘forerunner’ and ‘founder’ generations have different attitudes towards questions of disciplinarity and interdisciplinarity than those of the ‘follower’ generation (to borrow terminology from Ben-David and Collins, 1966). In this paper we expand on our earlier work (Lewis and Bartlett 2013) to show the ways in which the differences in cultural values at play in bioinformatics has an effect on the way in which bioinformaticians are (or are not) integrated – practically and ideologically – into the reward system of academics. This is a paper then about the way in which being in the middle can feel like being nowhere in particular.

While a glib definition of bioinformatics is that it is the combination of computing and biology, the boundaries around bioinformatics are fuzzy and ill-defined (Fenstermacher 2004; Lewis and Bartlett 2013; Stevens 2013). The establishment, by the United States (US) National Institutes of Health, of a ‘Bioinformatics Definition Committee’ (NIHBDC) provides an example of this definitional difficulty. Bioinformatics is a specialism that includes, among other activities, data management and data cleaning, data visualisation and data modelling, the statistical and computational analysis of large biological datasets, and the creation and application of new and existing algorithms. The committee caught a tremendous diversity of this work in its net, categorising bioinformatics as “any research, development, or application of computational tools and approaches for expanding the use of biological, medical, behavioural, or health data including those to acquire, store, organise, archive, analyse or visualise data” (Huerta *et al*. 2000: 1). A little over 15 years on and bioinformatics remains, intellectually, an interdisciplinary bricolage, incorporating experts and expertise from various disciplines. At the same time, its institutional position is equally ill-defined, finding itself occupying both the role of a cutting-edge science and that of a service provider subordinate to the other life sciences (Lewis and Bartlett 2013). The heterogeneous nature of the specialism undoubtedly makes studying the field difficult, especially examining the trans-national cultures of bioinformatics (see Salter *et al.* 2016). For the purpose of this research, we have opted for a similarly inclusive definition to the NIHBDC with regard to the types of activities it involves, but with a specific empirical focus on post-HGP academic bioinformatics in the UK.

The primary data the paper draws upon was derived from a 38 question web-based survey sent to a sample of approximately 1,000 UK academics working in and around the field of bioinformatics (2010–2011) during the ‘Adulthood’ phase (Ouzounis 2012). The Bristol Online Survey (BOS) tool was used to design the survey, which was distributed by email to the sample. 326 respondents completed the survey. Of those 326 respondents, 80.4% were male and 19.6% were female, with 10 providing no response to this question. Of those who responded to the question on job position, 25.6% described themselves as professors or readers, 22% were lecturers and senior lecturers, 27.8% categorised themselves as ‘researchers’ (in various forms), 11% were PhD students and 13.6% classified themselves as ‘other’, which included research officers, lab managers, technicians, developers and database curators. In the paper, we identify survey respondents by their professional status and their associated school or department.

The paper also draws on a qualitative study of genomic scientists working at 5 UK universities, which was conducted prior to the survey (2004–2009). 31 interviews were supplemented with ethnographic observations of conferences, workshops, bioinformatics courses, and informal meetings. Respondents were a mixture of mid and later career researchers, with the exception of two PhD students. A senior manager involved in the distribution of funding for bioinformatics research was also interviewed. The perspective of bioinformatics presented in this paper is therefore that of academic bioinformaticians, and of academics working in the field of bioinformatics, based in research-intensive universities. Overwhelmingly, the respondents were working in the particularly prominent branch of bioinformatics that deals with the analysis of data from post-HGP –omic (genomic, proteomic, etc.) life science research. Interviews and survey responses were analysed by content for emergent themes, with the ethnographic engagement of the authors being used to help analyse and understand the anonymous survey responses. Interview extracts used in this paper have been anonymized.

## The Space In-between: Biology and Computer Science

While previously we have discussed questions of *interests* and *power* within bioinformatics and between bioinformatics and its parent disciplines (Lewis and Bartlett 2013), this paper addresses questions of different disciplinary *values*. In particular, we discuss the ways in which different value systems – especially the reward systems – are reflected in the social structures of disciplines, leaving adrift those occupying the space in-between. Established ways of working in the ‘parent’ disciplines – whether computer science or biology - has meant that the interdisciplinary terrain of bioinformatics is troubled by competition and ignorance. Computer scientists, for example, are perceived as being *parasitic* on the field of biology (see Longo and Drazen 2016), while biologists are accused of not accepting computer scientists as conducting *worthwhile* research. What is deemed important in one ‘parent’ discipline may be regarded as trivial in another as different disciplines have different cultures with different *value* systems.

Bioinformatics encompasses a range of value systems as it sits on the borders of biology, computer science, and other specialisms such as medicine, statistics, etc. In some senses, it acts as a bridge between these established disciplines. This, however, places bioinformaticians outside settled boundaries, leading survey respondents in our research to bemoan the way in which the novelty of interdisciplinarity is treated by the academy. Typically, participants lay the blame at the organisational arrangements of higher education systems, which establish and affirm disciplinary differences. The socializing institution such as the university is arranged in ‘departments’ or equivalents, often with strong boundaries between them. Within them, there will be strong relations of subject-based identification and strong vertical (hierarchical) relations.

“The biggest challenge for University bioinformatics research is the existence of *discipline silos* resulting from departmental structures. Bioinformatics is very cross-disciplinary and has inputs from many fields. So, this necessarily results in the need for successful groups to have members containing expertise in different fields to coexist in the same department. Whereas, most departments focus on a topic (say maths, biology, or computer science)” [Senior Lecturer in Biology and Mathematics departments (survey)].

The description offered by the Senior Lecturer in Biology and Mathematics describes the academy consisting of disciplinary tribes and silos (see Becher and Trowler 2001). With scientists coming to bioinformatics from a number of different disciplines – and with the rise of dedicated bioinformatics programmes from within a nascent discipline of their own – the interests and affiliations of bioinformaticians are also a patchwork: there are boundaries within the space in-between as well (see Lewis and Bartlett 2013).

“Bioinformaticians tend to have a silo mentality and compete with each other instead of cooperating” [Professor in Biology department (survey)].

Interdisciplinary research groups have been found to be culturally and intellectually fragmented (Delamont, Atkinson and Parry 2000). Similarly, bioinformaticians present bioinformatics as a field riven by divisions. In some cases these silos appear as if impermeable, with little interaction between biologists and computer scientists who inhabit the space, and little recognition and understanding of each other’s disciplinary hinterlands (Lewis and Bartlett 2013). Indeed, when two cultures meet, the differences in their values are often rendered more visible (Inglehart and Baker 2000; Penders *et al.* 2015).

“There is a great deal of *ignorance* amongst biomedical vs math/comp sci[ence] sides as well as divisions in between. There is much hype about interdisciplinarity but the reality has a long way to go” [Lecturer in Bio-nanoengineering department (survey)].

Doing collaborative (or collective) interdisciplinarity is no easy task. Despite the epistemic centrality of the field in post-genome science, bioinformatics often finds itself occupying a peripheral position institutionally (Lewis and Bartlett 2013), being located on the edges, especially within the structures of reward and recognition. This is particularly the case when biologists and other life scientists from outside bioinformatics’ boundary position bioinformatics as a service, making the relationship between biologist and bioinformatician similar to that of scientist and technician.

“Finding collaborators willing to perform experiments to advance bioinformatics, rather than viewing bioinformatics as a modern form of a statistics service [is a challenge]” [Senior Lecturer Computer Science department (survey)].

Bioinformaticians may act as bridges between cultures, but they are also subject to the tensions between the interests – whether conceptual or material – of these cultures. Bioinformaticians complain that bioinformatics is incorporated into the established academy by way of the imaginations of those outside the field.

“Bioinformatics is not a discipline that is being funded as a discipline unless it is a computer science based type project… At the end of the day, research councils are controlled by biologists or medics. There are some bioinformaticists on these boards but at the same time they are not taking on board bioinformaticists like myself in need of funding bioinformatics-type research: not just computer science but to research bioinformatics approaches or strategies whether it be data analysis, it is all just statistics to them and it is just labelled that” [Associate Professor in Bioinformatics (interview)].

There are multiple dimensions involved in the heterogeneity of the field that is bounded by the term bioinformatics. Bioinformatics as computer science, it is argued, is treated as a discipline, whilst bioinformatics as data analysis is treated as infrastructural support. In the US, distinctions are also sometimes drawn between computational biology and bioinformatics. Computational biology – the study of biology using computationally designed techniques – is often defined as a science in its own right, while bioinformatics is sometimes positioned as being service orientated, involving the management of databases and the visualization of data (see Leonelli 2013). In our explorations of UK bioinformatics we have been told of the differences between bioinformaticists and bioinformaticians, another distinction between those involved in autonomous research-driven work and those who offer a service (see Lewis and Bartlett 2013). However, these different definitions of bioinformatics can be seen to be differences in value, and it is the values of biology and biologists that seem to matter. Understood as a service, bioinformatics is ordinary and unremarkable, with those practicing it unworthy of the rewards and recognition due to scientists doing creative and original work within disciplinary boundaries. The struggle over the characterisation of bioinformatics is therefore ongoing, with even those working within the field finding it difficult to get a grip on what it might contain.

“Bioinformatics needs to be more clearly defined, as different institutes appear to have different definitions of the term. Does it just concern molecular biology, or does it cover all areas of biology? How much of it relates to databasing and data format issues, and how much to other aspects of modelling?” [Reader in Biology department (survey)].

What we can say, though, is that as an interdisciplinary specialism, bioinformatics encompasses a variety of disciplinary outlooks, practices and expectations. Inside bioinformatics, participants describe a divided specialism, with little solidarity between the disciplinary camps and with each group ignorant of the other’s practices and parent discipline.

“The difficulties lie with the medics/biologist[s] who do not appreciate the intellectual challenge of the various problems thrown up. They have, for example, rarely any understanding of the many problems with the interpretation of micro-array data. They believe that they have extensive samples, when in fact they often have only a small sample of very high dimensional data. They… also believe in data mining without proper validation” [Emeritus Professor in Mathematics department (survey)].

And:

“There is some resistance to sharing based on the view of bioinformaticians as parasites on hard-working biologists” [Research Associate in Bioinformatics research centre (survey)].

While biologists are criticised for their failure to understand the statistical challenges involved in the field, computer scientists are seen as freeloaders or leaches subverting biology in the pursuit of interests that are alien to biologists. Of course, such differences can be explained as knowledge deficits. But these kinds of differences are not solely questions of ignorance or expertise; they are not just about the subject matter of disciplines. It is not just that biologists do not understand statistics and computer programming, and that computer scientists do not understand the laboratory techniques behind the Genome Wide Association Studies (GWAS) data they analyse, or the biological nature of DNA sequence variations. They also do not understand one another’s research *culture*.

“There is also a lack of knowledge of biomedical research culture, on the part of computer scientists. There are fundamental differences in the logic of research in the two fields” [Post-doctoral researcher in Computer Science (survey)].

Disciplinary cultures, of course, are more than just knowledge and practice. Culture also involves values (see Knorr-Cetina 1999). The tensions at work in bioinformatics and the problems of collective or collaborative interdisciplinarity (Calvert 2010) are also about *values* – what is deemed *worthwhile*, what sort of knowledge and work is valued, and what *deserves* reward and recognition.

## Different Value Systems in Bioinformatics

Values are more-or-less taken for granted when work is unidisciplinary. Everyone involved has, to some degree, internalised the values of the discipline to which they belong. It is in the poorly defined borderlands where cultures, and values, clash and where these values are made explicit. As we have described, bioinformatics is a hybrid discipline made up of various specialisms with different perspectives (Lewis and Bartlett 2013; Stevens 2013). What is considered worthwhile in one specialism might not be considered so in another.

“With my very cynical hat on I would actually say that everybody is using bioinformatics as ‘oh look there is something else I can get some funding to work on my pet technique’, but nobody is ever thinking about what it is achieving? Let’s say for instance we are supposedly solving these biological problems and all these sorts of things. But the computer scientists come in and it is more of the case of here is a problem that I can apply my pet technique that I have been working on in the past ten years. Oh I can get some money to work on it but it doesn’t have to actually have to produce anything useful” [Lecturer in Biology (interview)].

In reality, it is extremely difficult to distinguish cognitive and practical differences from value differences as they are all part of any discipline’s socialisation. In this interview extract – the biologist by training – is unable to see the *value* in work driven by the interests of computer scientists. What this illustrates is that computer scientists and biologists *are* more than cognitively distinct; they are culturally different too. They have different value systems when it comes to understanding the purpose and nature of scientific work. The symbolic boundaries around intellectual fields have major implications for the self-identity of specialists; socialising the kinds of activities and behaviours that should be valued and valorised. Respondents in our research are alert to this cultural mismatch.

“[The biggest challenge is] understanding each other’s problems, requirements, difficulties and backgrounds” [Research Fellow in Computer Science (survey)].

While normal academic disciplines are made up of like-minded members or what Knorr-Cetina calls ‘epistemic cultures’ (Knorr-Cetina 1999), bioinformatics, as a true interdiscipline, is not. If we look at the same question from the perspective of both computer scientists and biologists we might find that the view is markedly different. For a biologist working inside bioinformatics’ boundary, a bioinformatics that does not adopt the values of biology is inconsequential.

“To be good, bioinformatics has to be relevant to biology and answer real, testable questions. Too much bioinformatics is irrelevant and can be ignored” [Research Fellow in Genetics department (survey)].

For computer scientists working in bioinformatics, biologists are ignorant of the very nature of computational ‘dry’ laboratory work.

“[The challenge is] overcoming the attitude of many biologists that anything that requires computation is not really biology; changing the perception that bioinformaticians are support personnel” [Research Fellow in Computer Science department (survey)].

Much writing on the problems of interdisciplinarity is concerned with making tacit knowledge explicit; this is often only constructed in practical and cognitive terms – for example, the correct way to conduct an experiment, or discussions over what constitutes an enzyme. However, tacit and understated differences between cultures and values also need to be reconciled if the path of interdisciplinary research is to be a smooth one. Wet laboratory scientists are different to dry laboratory scientists – they work with different data, produce different kinds of outputs and see value in different ways of working. Biologists in the wet lab might be interested in how they can get their experiment to run successfully, whilst computer scientists in the dry lab might be concerned with programming or modelling (see Delamont *et al.* 2000). These apparently practical differences are intimately connected with the value system of the discipline. For example, differences in values have the result that some bioinformaticians maintain that their work is not seen as worthwhile or ‘real’ by biologists.

“Biologists often consider bioinformatics as being ‘made up’ or somehow not real […] I was told that I had to do real experiments to be successful despite the fact that my bioinformatics work had led to several *Nature*/*Science* publications” [Scientific Curator in Bioinformatics research centre (survey)].

Universities are arranged around established scholarly disciplines such as biology, maths and computer science; nature (and some research) is not. To achieve the goals of interdisciplinarity many different obstacles must be overcome and chief among these are conventional institutional arrangement that inhibit or prevent such endeavours. Bioinformatics is often presented as being at odds with the reproduction of discipline-specific research. As a phenomenon that sits uncomfortably without a coherent ‘pure’ classification, bioinformatics not only troubles research scientists working in and around the field; it also disrupts the traditional pattern of the academy.

“Whatever organisations say, they do not like multi-disciplinary research. In cross[ing] boundaries, you confuse people, and confuse systems. Worst, you get expected to sit on two or three committees. Finally, everybody thin[k]s that their work is harder than everyone else’s. So, if you work in two or three disciplines, people generally assume that your work is simple – you aren’t doing real computer science of course, you aren’t doing real biology” [Reader in Computer Science department (survey)].

Interdisciplinary work such as bioinformatics confuses the established system, which in turn becomes a problem for scientists who inhabit those spaces. The academy finds it difficult to accommodate boundary crossers, rendering those with individual interdisciplinarity (see Calvert 2010) anomalous. The survey respondent explains how they have to contribute to multiple committees across different departments. The result is that their work, rather than being recognised as cutting-edge and interdisciplinary, is [*mis*]understood as simple, routine and/or trivial. While bioinformaticians are not seen as ‘pollutants’, Douglas’s work on ‘matter out of place’ can offer us some insights here. Douglas’s analysis of pollution emphasizes the cultural significance of symbolic boundaries that define cultural categories, and so keep distinct the ‘natural types’ that constitute any given cosmology or cultural domain (Douglas 1966). In Douglas’s analysis, phenomena that appear to transgress such symbolic boundaries or are hybrid, ambiguous types, become treated as anomalies. The underlying model of discipline-based academic work is, in Bernstein’s (1971) terms, based on strong principles of classification that define not only the ‘cosmology’ of knowledge-domains, but also concrete organisational arrangements. Bioinformatics – as an anomalous field – is therefore organisationally and institutionally sidelined.

“Bioinformatics… is viewed as IT by biologists whether it be [through a] lack of understanding or whatever. Which is probably right, actually, because if you talk to biologists about an area that they are not familiar with, whether it be another area of genetics or whatever, they won’t want to talk about it. They either rubbish it or won’t carry on with the conversation. That applies for bioinformatics because they don’t understand it. It is either statistics to them or it is computer science and in that sense it hasn’t been accepted as a discipline in its own right by biologists and funders and has ended up as a service” [Professor in Bioinformatics (interview)].

What is the effect of being in-between these different value systems on the scientists occupying that space? Rewards systems validate the *value* system of the dominant or host groups. Bioinformatics, as an anomalous immigrant, which values different ways of working and different products of scientific work, does not fully partake in the reward systems of biology. Although home to many different kinds of work, which may affect how different personnel are rewarded, fundamentally, bioinformatics is positioned by biologists as a service to biology, as are all the field’s inhabitants – whether they are integrating, managing and applying existing programmes and databases or advancing computational method development – with all the repercussions this has on being valued by the academy.

## What is Valued? Credit, Reward and Esteem in Bioinformatics

The division of labour involved in big biological projects, of which bioinformatics is a key component, depends on high degrees of trust and mutual recognition. Rewards and recognition in collaborative, interdisciplinary projects can come at the personal, group, and institutional level. Indeed, there are multiple types of contribution to research efforts for which people might wish to receive credit, with different disciplines valorising different types of contribution and outputs. These value systems are then reflected in the social structures – especially the reward structures – of the ‘parent’ disciplines.

“The other thing that causes [a] problem […] is that biologists publish papers in journals and that is how they get credit. Computer scientists get credit for invited talks at computer science conference[s]…Whereas a biologist will have first author papers, computer scientists would have a series articles which have appeared at the proceedings of conferences and that is how they value each other. Bioinformaticists measure their worth in terms of open ware software, which is released to the rest of the community. That means if you are sitting there as a biology head of department or whatever and you have gone and got yourself a multi-disciplinary team, half of them are going to be producing outputs that your biological community is going to regard as completely worthless. So, you have got a whole range of cultural change issues which are to do with the discipline silos and how they move about” [Senior Manager funding Biotechnology (interview)].

Bioinformatics, once more, is described as being made up of (at least) two cultures that valorise different things, but which must learn to live not just side-by-side but together. Bioinformatics’ location of being an in-between discipline positions bioinformaticians as anomalous with regard to the cultural measures of value. Valorised scientific ‘outputs’, not just knowledge domains, are defined by discipline. Thus, despite some scientists moving into the specialism, the fact that they are rewarded as biologists or as computer scientists, etc., creates mixed messages for those scientists colonising a new territory. Given that most panels that fund bioinformatics work are made up of biologists this has a significant effect on those in the field.

“[The] main issue is that the methods for recognizing credit for software are not widely accepted as many hard-core biologists (and Research Councils) do not recognize these. Same applies to the submission of datasets to international repositories” [Professor in Bioinformatics department (survey)].

Bioinformatics’ status as an interdiscipline *diminishes* the respect with which it is held, despite the fashionable lauding of interdisciplinarity. The virtues of its work are not understood, judged to be worthless, or worse still, the work is incorporated into the structure of the academy as a service subordinate to established disciplines. Douglas (1966) tells us there are several ways to treat anomalies and un-naturals. “Negatively, we can *ignore*, just *not perceive* them, or perceiving we can *condemn*. Positively we can deliberately confront the anomaly and try to create a new pattern of reality in which it has a place” (p. 38). For bioinformaticians in our studies, the specialism of bioinformatics is often not recognised or simply ignored as a site of serious of research.

“Various UK funding bodies are ignorant of the special needs of working at a discipline interface – their panels are parochial and tend to dismiss proposals as either not exciting biology or not exciting computer science or both, and/or not research” [Professor in Biology department (survey)].

Classically, institutional hierarchies and funding panels are set up to value the working practices of established disciplines. They are accused of insufficient understanding when it comes to judging those working in an interdiscipline.

“Administrators do not distinguish between bioinformatics as research [or] as support; many do not distinguish us from technicians” [Professor in Biology department (survey)].

Despite bioinformatics encompassing a range of activities – some more academic than others – the complaint is that everyone working in the specialism is positioned as service personnel. Even when bioinformatics is afforded the status of a disciplinary field, it has to fit into established disciplinary borders and their attendant value systems, or remain an unassimilated immigrant that continues to draw its values from the culture of the home discipline.

“I am member of two departments [at a] university, mathematical sciences and biological sciences. For RAE/REF purposes, I am classed as a biologist” [Senior Lecturer in Biology and Mathematics department (survey)].

The positioning of the field structures the principles of credit, reward and esteem at the personal level. The hybrid nature of bioinformatics as an interdiscipline invariably affects those scientists that work in the field. In a similar way to how traditional disciplinary domains feed into the academic systems of reward and recognition, so the way bioinformatics is configured by the academy as a service, or junior collaborator at best, has repercussions for bioinformaticians, whether that is in terms of obtaining their own funding – and therefore much valued scientific autonomy – or the credit they are accorded for their contribution to research.

“[The biggest challenge is] getting first authorship on papers where you have collaborated with an experimentalist” [Post-doctoral researcher in Bioinformatics department (survey)].

Scholars of disparity and disproportion have extensively researched the distribution of status and value (see Abbott 1981; Fraser 1997; Collins 2004). Classically, science and technology studies scholars have also written about value and reward structures in scientific disciplines (see, for example, Shrum 1984; Watson and Meiksins 1991; Gunnarsdottir 2005). One of the common threads in the work on scientific specialisms is that the assignment of authorship is a significant process in which academics’ work is recognised as valuable or not. In the traditional, small science model of research, individual researchers or a handful of collaborators often took responsibility for entire research projects. They designed their own experiments, and collected and analysed their own data (Birnholtz 2006). In big science, very rarely is the scientific paper written by a single author. Nowadays, the trend is towards multiple authors (Galison and Hevly 1992), and in the big life sciences there can be hundreds of authors on a single paper. These developments have created, in Beaver’s (2001) words, ‘fractional scientists’. The position that the author is assigned often reflects their level of contribution to the paper, with first and last author being the most significant. Survey respondents complain of being punished for conducting interdisciplinary work.

“On the one hand, we are told that we should work in interdisciplinary teams; on the other hand, for RAE/REF and/or promotions, only first or last authorship on papers are considered. Can’t have it both ways!” [Senior Lecturer in Systems Biology department (survey)].

According to Klein (1996), crossing boundaries is a defining characteristic of our age. Immigrants can bring fresh insights and ways of working (Brewer 1999). However, Calvert (2010) describes the way in which UK evaluation procedures – such as the REF – that tend to work within established disciplinary boundaries, runs counter to interdisciplinary research. As an interdiscipline, bioinformatics lies in the interstices between biology and computer science. This invariably means that bioinformaticians occupy a liminal space in the academy. ‘Middle-ness’ can be understood as centrality – in the sense that bioinformatics is a fundamental and indispensable aspect of post-HGP life science. But as we have seen, middle-ness can also mean ‘in-betweenness’ – occupying an anomalous and ambiguous position. How can the ‘economy’ of science reward those who do not fit into the established categories?

“Much greater respect for significant middle author contribution needs to be afforded. Again, this impacts on many fields, not just bioinformatics” [Senior Lecturer in Systems Biology department (survey)].

Ambiguity as to what bioinformatics is and the inherent ‘middleness’ of interdisciplinarity has meant that bioinformatics, like other interspecialisms, suffers from being branded a conduit, or a bridge. Bridges connect, but they are also walked over. Interdisciplinary scientists are at odds with pure, traditional disciplinary categories or are still in transition towards a coherent, established identity. This is then reflected in their contribution as middle authors or junior collaborators, bridging fragmented disciplines with different value systems. Expertise in big biology cannot be centralised because no one person can possess the expertise required to produce and analyse big biological data – each scientist takes on a narrowly specialised role within a project. Therefore, because expertise and work is distributed, rewards and recognition are also distributed. Rather than being rewarded as first authors, bioinformaticians often bemoan being positioned ‘in the middle’ – as fractional scientists – tying and bringing together research rather than leading and directing it; or, worse still, they are not recognised at all.

“The researchers I do the work for ‘forget’ that I made key - if small - contributions to the work and leave me off the research paper. It is surprising how so many intelligent people can act so dumb when I remind them of their omission” [Research Fellow in Biology department (survey)].

Of course, bioinformaticians are not alone in this [dis]regard. Statisticians, whose analysis is often central to the claims of a research paper, often find that they are not ‘authors’ when the paper is published (Parker and Berman 1998). Just as with bioinformaticians, the published articles are not ‘statistics’ papers. Rather, they make contributions to, for example, biology papers. Interdisciplinarity therefore is presented as being at odds with the institutionalised systems of reward and recognition, despite the fact that the rhetoric of interdisciplinarity as a good in and of itself is frequently deployed by funders and other research planners and administrators. This puts bioinformaticians in an unenviable position.

“Balancing service and research, and establishing a model which allows the PI to get funding and sustain a lab, whilst continuing to publish in both biologically focused and technique/informatic-focused journals [is a challenge]… Heads of Schools are asking for evidence for RAE/REF etc of 4 PI papers and grants which are hard to come by for some bioinformatics researchers” [Professor in Biology department (survey)].

Scientific cultures are built on an economy of reputation (Whitley 2000). Although claims to the material ownership of knowledge – patents, copyright, commercialisation as academic ‘impact’, etc. – grow as universities embrace their supposed position as entrepreneurial engines of a ‘knowledge economy’ (see Gibbons *et al.* 1994), scientists have traditionally been concerned with recognition – claims of priority and eponymity (for the classic treatment, see, of course, Merton 1968). Authorship, especially first (and in the life sciences, last) authorship, is the currency of most academic careers. Funding agencies and universities are more likely to support scientists with proven track-records of successful research measured by way of authorship of publications in high ‘impact factor’ journals. Research papers are ‘capital’ which scientists can parlay into material support, therefore claiming territories of research. Not everyone, though, who contributes to research is equally rewarded in this way, or even rewarded commensurate to their efforts. It was ever thus. Writing about the age of the gentleman scientist, Shapin (1989) emphasises the role that technicians played in the great discoveries of the past, yet in the publications and official histories these technicians were rendered ‘invisible’. In a similar vein, bioinformaticians complain that being seen as providing a service due to their anomalous status of being in-between has meant they too were either sometimes ‘forgotten’ when it came to the authorship of papers, or viewed as factional scientists, they get lost in middle authorship.

## Conclusion

This paper outlines some of the ways in which bioinformaticians, as interdisciplinary scientists, find themselves in the space in-between the established disciplinary domains of biology and computer science. There is a great deal of policy pressure, especially in biology, for the promotion of interdisciplinary research (Strathern 2006). Interdisciplinarity is hailed as being a key contributor to contemporary scientific breakthroughs (Hollingsworth and Hollingsworth 2000) and a driver for innovation (Gibbons *et al.* 1994). The top down pressure has seen the creation of hybrid (or fractional) scientists, part biologist, part computer scientist, who are able to cross established disciplinary boundaries. This movement can upset the culture and organisation of an intellectual field.

There are boundaries between biologists and bioinformaticians as well as boundaries between biologists and computer scientists within the space itself. People, or groups of people, that transgress boundaries, or occupy a position in-between categories, also occupy an anomalous or ambiguous cultural and institutional space, which in turn creates problems for an ‘economy’ based on unidisciplinary esteem and recognition. These interdisciplinary scientists are being integrated into the academic order, but the ways in which they are being accommodated are shaping their forms of academic credit and esteem. Currently, biologists hold the upper hand in UK bioinformatics collaborations, they control most of the funding, are the producers of the primary inscriptions (Lewis and Bartlett 2013), and constitute the team leaders for most projects. The result has meant that as in-between scientists, participants in our research complain of the ways in which their contributions to the work of post-HGP life science are taken for granted, not valued, overlooked, or, worse, the very legitimacy of their research programmes has been questioned. When they are valued and credited with authorship, respondents in our survey bemoan the weak position they occupy within the list of authors and how this diminishes the academic capital that they are able to claim. Bioinformaticians might therefore not be as invisible as Shapin’s technicians (or, indeed, as invisible as the laboratory technicians of contemporary life science), but they are rarely afforded the centre stage. Instead, they are ‘hidden’ in the middle.

Of course, UK academia is going through a period of change as traditional ways of working are being eclipsed or superseded (see Gibbons *et al.* 1994). In some cases, values from industry are infiltrating academic ones (Kleinman and Vallas 2001), which might extend to different ideas of reward and success. Furthermore, Wadmann (2014) has shown how contract research can be exploited in order to create the autonomy required to pursue one’s own research agenda. However, this does not get away from the fact that esteem and recognition are central to scientists’ identity and the trajectory of their careers. Without value or recognition for their work in a form accepted by the established disciplines – and in the life sciences this currently means prominent authorship – scientists such as bioinformaticians working under a big science model might find that in the future they are even more dependent on the leaders of their team to secure funding. The shape and culture of this new field therefore is still to be settled, with founders and followers with radically different value systems colonising the territory (Bartlett, Lewis and Williams 2016). More, the imaginations of powerful actors outside the field also help configure the territory. This leaves us questions for the future: will bioinformatics, to continue the metaphor for a moment, be a colony dedicated to providing a service to the parent discipline (of biology), or will it be an independent ‘nation’ in its own right? The resolution of these struggles will determine the economy of this new territory – whether it be publications or computer programmes that are valorised, or more fundamentally, whether it be a domain of technicians or one of scientists.
